# A framework for stakeholder engagement in the adoption of new anti-malarial treatments in Africa: a case study of Nigeria

**DOI:** 10.1186/s12936-023-04622-2

**Published:** 2023-06-17

**Authors:** Olugbenga Ayodeji Mokuolu, Oladimeji Akeem Bolarinwa, Oluwatumobi Racheal Opadiran, Hafsat Abolore Ameen, Mehul Dhorda, Phaik Yeong Cheah, Chanaki Amaratunga, Freek de Haan, Paulina Tindana, Arjen M. Dondorp

**Affiliations:** 1 Department of Paediatrics, University of Ilorin, Ilorin, Nigeria; 2 Department of Epidemiology and Community Health, University of Ilorin, Ilorin, Nigeria; 3 Messentia Medicare, River Park Estate, Abuja, Nigeria; 4 Mahidol Oxford Tropical Medicine Research Unit, Mahidol University, Bangkok, Thailand; 5 Centre for Tropical Medicine and Global Health, Nuffield Department of Medicine, University of Oxford, Oxford, UK; 6 Copernicus Institute of Sustainable Development, Utrecht University, Utrecht, the Netherlands; 7 Department of Health Policy, Planning and Management, School of Public Health, College of Health Sciences, University of Ghana, Accra, Ghana; 8 The Ethox Centre, Nuffield Department of Population Health, University of Oxford, Oxford, UK

**Keywords:** Artemisinin resistance, Stakeholder engagement, Artemisinin-based combination therapy, Framework, Antimalarial treatment policy

## Abstract

**Background:**

Recent reports of artemisinin partial resistance from Rwanda and Uganda are worrisome and suggest a future policy change to adopt new anti-malarials. This is a case study on the evolution, adoption, and implementation of new anti-malarial treatment policies in Nigeria. The main objective is to provide perspectives to enhance the future uptake of new anti-malarials, with an emphasis on stakeholder engagement strategies.

**Methods:**

This case study is based on an analysis of policy documents and stakeholders’ perspectives drawn from an empirical study conducted in Nigeria, 2019–2020. A mixed methods approach was adopted, including historical accounts, review of programme and policy documents, and 33 qualitative in-depth interviews and 6 focus group discussions.

**Results:**

Based on policy documents reviewed, the adoption of artemisinin-based combination therapy (ACT) in Nigeria was swift due to political will, funding and support from global developmental partners. However, the implementation of ACT was met with resistance from suppliers, distributors, prescribers, and end-users, attributed to market dynamics, costs and inadequate stakeholder engagement. Deployment of ACT in Nigeria witnessed increased developmental partner support, robust data generation, ACT case-management strengthening and evidence on anti-malarial use in severe malaria and antenatal care management. A framework for effective stakeholder engagement for the future adoption of new anti-malarial treatment strategies was proposed. The framework covers the pathway from generating evidence on drug efficacy, safety and uptake; to making treatment accessible and affordable to end-users. It addresses which stakeholders to engage with and the content of engagement strategies with key stakeholders at different levels of the transition process.

**Conclusion:**

Early and staged engagement of stakeholders from global bodies to community level end-users is critical to the successful adoption and uptake of new anti-malarial treatment policies. A framework for these engagements was proposed as a contribution to enhancing the uptake of future anti-malarial strategies.

## Background

Translation of scientific evidence into policies and interventions is not always straightforward or swift [1]. One major example is the problematic introduction of artemisinin-based combination therapy (ACT) as a new generation of anti-malarial therapies in the late 1990s, when all conventional anti-malarial monotherapies including chloroquine and sulfadoxine-pyrimethamine (SP) were failing globally due to multidrug resistance [2]. Expert meetings were conducted at the World Health Organization (WHO) to review evidence, culminating in policy recommendations towards the adoption of new treatment regimens in malaria-endemic regions. The experience over the years, however, indicates that neither scientific evidence nor WHO recommendations were sufficient to realize the effective adoption, implementation, deployment and uptake of anti-malarial treatment policies [3–5].

There are numerous other drivers, often unique to individual countries, that influence the adoption of anti-malarial treatment policies [5]. One major driver is the impact of country-level stakeholders’ engagement [6]. Operationalizing evidence into practice does not end with policymakers; engagement with all stakeholders (e.g. regulators, suppliers, prescribers and end-users) starting from early stages of evidence generation to the final stage of uptake is central. [7, 8]. This manuscript is predicated on current evidence from Southeast Asia [9] regarding resistance to artemisinin and partner drugs with resultant failure of ACT, and the recent reports of artemisinin partial resistance from three African countries [10–13]. Widespread artemisinin resistance in African countries could lead to a rise in the disease burden with devastating impact on mortality similar to events in the 1990s [14].

Artesunate-amodiaquine (ASAQ) and artemether-lumefantrine (AL) remain efficacious for treatment of uncomplicated *Plasmodium falciparum* malaria in Nigeria and most African regions [15–17]. There are however, reports of ACT failure reported from Burkina Faso, Angola and the Democratic Republic of Congo, which are debated [18–22]. New classes of anti-malarial therapies are being developed [23, 24], but they are at least 5 years away from market introduction [24, 25]. The WHO has recently proposed new strategies to address anti-malarial drug resistance in Africa, which include better leveraging existing tools to preserve the therapeutic life of current artemisinin-based combinations until other viable solutions become available [13]. Suggestions include exploring the potential of rotating artemisinin-based combinations before high treatment failure rates are detected, deploying multiple first-line therapies at the same time, and extending the duration of treatment regimens [13]. Additionally, triple artemisinin-based combination therapy (TACT), where artemisinin is combined with two carefully selected, widely-used partner drugs is also proposed and being investigated as a possible strategy to prevent or delay artemisinin resistance from emerging [25–30]. All these strategies will require policy change because most endemic countries already have operational anti-malarial policies and guidelines. The context of the policy change may be more complex depending on the type of treatment, required delivery methods, and health system variabilities among others.

To aid this potential new transition, lessons learnt during previous anti-malarial drug transitions are discussed here. It is envisioned that these lessons learned and related best practices will inform future policy change in terms of how to more efficiently engage stakeholders to adopt and implement new anti-malarials. Therefore, this study was conducted to review the evolution and adoption of anti-malarial treatment policy processes and the change of anti-malarial implementation processes in Nigeria with the objective of providing perspectives that will enhance future uptake of new anti-malarial treatments or treatment strategies in an age of artemisinin resistance.

## Methods

This study is based on two approaches.

1. An analysis of stakeholders’ perspectives extracted from a qualitative study conducted in Nigeria between December 2019 and June 2020 involving 33 in-depth interviews (IDI) and 6 focus group discussions (FGDs) [31].
2. An analysis of programme, and policy documents and two further interviews conducted in December 2021 to include historical accounts with key informants immersed in the malaria elimination program in Nigeria.

A mixed methods was adopted for this study. This involves qualitative (IDIs, key informant interviews and FGDs) data obtained from major malaria stakeholders in Nigeria, including policy makers, regulators, manufacturers/distributors, prescribers, researchers, and end-users at community level and review of documents.

### Qualitative study

The qualitative phase of the study conducted with purposively selected respondents in both the federal capital city of Nigeria, Abuja and in a North-central State, Kwara, has been described previously [26, 27, 31]. For this case study, information from the 33 IDIs and 6 FGDs with key stakeholders (Table 1) was extracted. The study combined narrative and phenomenological strategies in qualitative enquiry. A more elaborate explanation of the respondent selection is reported elsewhere [26]

**Table 1:** Summary of IDIs and FGDs

Stakeholder group	Respondent interviewed	Number interviewed	IDI/FDG
Policy	National regulatory authority officials	5	IDI
	State malaria control program officials	4	IDI
Regulatory	NAFDAC official	1	IDI
	NMEP private sector desk	1	IDI
Distributor	Public sector drug wholesalers/distributors	4	IDI
	Private sector wholesalers/traders	4	IDI
Health service providers	Public sector: clinicians, pharmacists	5	IDI
	Private sector: clinicians, nurses, pharmacists, drug store	6	IDI
	Village health workers	2	FGD
Malaria Experts/Researchers	Immersed experts in Nigeria NMEP	2	Key informant
End users	Parents / caregivers	2	FGD
	Parents/ caregivers	3	IDI
	Community leaders	2	FGD

*NAFDAC* National Agency for Food and Drug Administration and Control, *NMEP* National Malaria Elimination Programme

### Key informant interviews

Two in-person interviews (~ 2 h each) were conducted with key informants affiliated to the malaria elimination program in Nigeria. There were further rounds of interviews with one of the key informants for further clarification and information via phone calls. The key informants were malaria experts and researchers whose experience spanned over 25 years in malaria control in the country. The interviews were tape-recorded to secure an accurate account of the conversations and avoid data loss.

### Document analysis

To trace the evolution and adoption of anti-malarial treatment policy processes and the change of implementation processes, the search data sources include online and screened programmatic, malaria treatment guidelines and policy-papers for relevance to anti-malarial policy change adoption. Boolean operators were used to narrow the search to the relevant documents from PubMed, African Journal online, Federal Ministry of Health website, WHO websites and Malaria Elimination Programme websites. The keywords used for the search were *antimalarial, policy, stakeholders’ engagement*, *artemisinin combination therapy, resistance, therapeutic efficacy* and *Nigeria.* Of the 50 documents identified, 9 were relevant to the evolution, adoption and implementation of anti-malarial policy and thereby included in the analysis. The findings from the policy document review, relevant qualitative data from IDIs, FGDs and expert interviews were combined to assess stakeholders’ engagement in the adoption of new anti-malarial treatment strategies in Nigeria.

### Study setting

#### Nigeria malaria control architecture

Nigeria has a population of over 200 million and the highest burden of malaria infections in Africa [32]. The Federal Ministry of Health (FMoH) through the National Coordinator is responsible for malaria control and elimination activities in the country [33]. The National Food and Drug Administration Control (NAFDAC) is responsible for regulatory function of malaria drugs and commodities. The Pharmaceuticals Manufacturing Group (PMG) plays an important role in the production of malaria commodities either independently or serving as franchise for local companies. The Nigerian health system operates a three-tier arrangement consisting of the Federal, State and Local authorities. The federal level formulates policy and controls the tertiary care. The states and local government levels are responsible for implementation of the policy. They are also responsible for regulatory, and implementation of activities related to secondary and primary levels of care respectively. Malaria service delivery, especially case management is channeled through public community and private systems. Health insurance coverage is low. Provision of anti-malarials in public primary healthcare facilities is largely free or heavily subsidized while the private system is largely fee for service except few enlisted in health insurance schemes [34]. Doctors are the primary prescribers in the tertiary facilities and to a large extent in the secondary levels [34]. Various other health professionals serve as prescribers in some secondary facilities and mostly in primary health care facilities. There is also a large informal system consisting of Proprietary Patent Medicine Vendors (PPMVs) and Community Pharmacists as prescribers. The roles and responsibilities of the prescribers are well enumerated in the Nigeria malaria control policy [35]. The private prescribers as well as informal sectors are guided to prescribe and dispense anti-malarials at controlled price including regulated brands as approved by the regulatory agency (NAFDAC). The prescribers at public primary and secondary level of care are routinely trained and updated on the malaria treatment guidelines in addition to notification and reporting.

#### Malaria burden and treatment policy in Nigeria

Nigeria, with about 63million annual cases of malaria, accounts for the largest burden of malaria globally; 26.8% and over 31.9% of the 241 million global malaria disease case and 627,000 deaths respectively [36]. The incidence of malaria in Nigeria reduced from 373 per thousand in 2010 to 314 in 2020 [37, 38]. The prevalence from Malaria Indicator Surveys in children 2–10 years has shown a decline from 42% [39] in 2010 to 23% in 2018 [37]. The current National Malaria Strategic Plan (NMSP 2021–25) has adopted a stratification approach to tailor actions in relation to peculiar characteristics of malaria within the various geo-political zones of the country. Since 2004, the treatment policy of malaria in Nigeria evolved from monotherapies to ACT [40]. Additionally, injectable artesunate for severe malaria and chemo-preventive strategies involving the use of intermittent preventive treatments in pregnancy (IPT) and seasonal malaria chemoprevention (SMC) were also adopted [40]. Thus, the country provides a rich experience of different cycles of translational policies on anti-malarials for key lessons and adoption of best practices.

## Results and discussion

### Stakeholder engagement in adoption of anti-malarial policy change

Due to the spread of resistance to anti-malarial monotherapies in the 1990s, the WHO, commissioned a review of literature in which evidence on resistance to chloroquine and other monotherapies was collected and assessed [2, 34]. These meetings resulted in the WHO recommendation to switch to ACT as global first-line therapy for the treatment of uncomplicated falciparum malaria [41]. Although most endemic countries followed this recommendation and adopted ACT in their national guidelines, significant delays were experienced between updated guidelines and the actual implementation [42, 43].

Individual countries usually appraise the recommendations of the WHO to reconsider their country-level strategies. Changing first-line therapies however involves a wide range of stakeholders at different levels of the healthcare system [5]. Stakeholder engagement in health policy is therefore critical for translating evidence into policy and implementation [44, 45]. An immersed expert recounted”*… a major challenge in the adoption of change in monotherapy was that the scope of stakeholder engagement was often not well-defined and supported by evidence. Furthermore, tailoring the stakeholder engagement strategies based on learning or analysis of the various stakeholders’ audiences are not well described.” (Interview Expert2)* When stakeholder engagement is not well coordinated the messaging also becomes fragmented and unclear [44, 45].

**Fig. 1:**
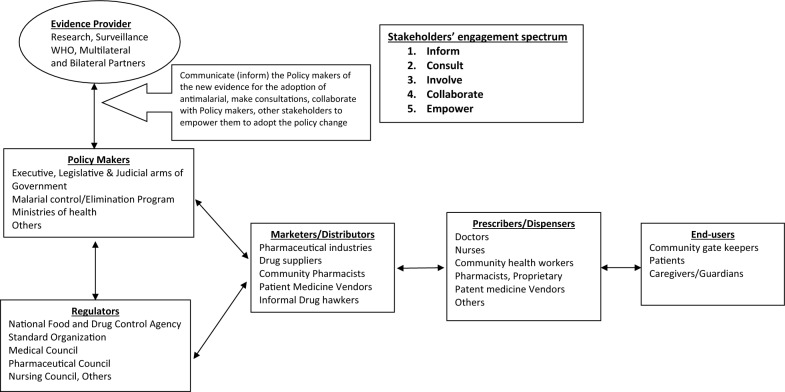
Framework for Stakeholders’ engagement in new antimalarial adoption

From the case scenario, a framework (Fig. 1) was developed for stakeholder engagement in the introduction and deployment of new anti-malarials or alternative strategies to treat malaria. This framework depicts the interrelations of the stakeholders ranging from those who generate evidence on anti-malarial therapeutic efficacy at local and international levels down to the anti-malarial end-users. The stakeholders include health policy makers, regulatory agencies, distributors, marketers and prescribers (Fig. 1). Five pillars of International Association of Public Participation (IAP2) [46] was employed to synthesize stakeholder engagement spectra to guide stakeholder engagement of new anti-malarials or treatment strategies.

### Evolution of anti-malarial policy adoption in Nigeria

#### Monotherapy era of pre-2001

Policy documents revealed a historical account of the evolution and adoption of anti-malarial treatment policy processes in Nigeria [33, 40, 41, 47]. These reviews showed an evolution in treatment policies during the era of anti-malarial monotherapies. Chloroquine provided relative stability in the use of monotherapy. An immersed expert recalled earliest guidance by the WHO on selection of anti-malarials recommending a four-point decision scale in the programmatic deployment of anti-malarials *‘‘…using these scales; antimalarial treatment failure rate* < *5% Grace, 6–15%, alert, 16–24% action stage which meant that there is need for identification of replacement molecule. A change was mandated when failure rate is* > *25% and the antimalaria molecule should be replaced with another that is more efficacious.” (Interview Expert1).*

In Nigeria, efficacy of chloroquine was prolonged despite resistance reported from Southeast Asia a decade earlier [40]. However, from 1988, efficacy in Nigeria declined below 70% [48]. Despite the reduced efficacy of chloroquine, the Nigerian Malaria Control Programme (as it was then called) waited another decade before reacting. Expert interview conducted revealed that this was related to limited awareness of the WHO policy guidelines by national policy makers. The two experts interviewed opined that an additional factor was the popularity of chloroquine among the end-users, which encourage continuous supply and demand of the drug. There was also perceived concern of the prescribers and experts about the ability of sulfadoxine-pyrimethamine (SP), which was the most viable alternative available, to withstand the same pressure as chloroquine before widespread resistance occurs. Buttressing these fears, was evidence of increasing SP resistance from East Africa, where SP had earlier been adopted as the first-line therapy [40]. While the country was in a dilemma, global discussion on the introduction of ACT began. After the WHO provided evidence on drug resistance to chloroquine, Nigeria malaria control programme in early 2000 was tending towards adopting SP, the only available monotherapy, as their national first-line anti-malarial therapy.

#### Transition era from monotherapy to ACT policy

The transition from monotherapy to ACT policy was not a swift process. One major approach to addressing the challenge of compliance was that of repackaging the existing monotherapies as age-group formulations. A request that required the Pharmaceutical Manufacturing Group members to retool their machineries. An immersed expert explained* “…until Nigeria engaged in the conversation of ACT adoption, the country mainly reacted to the growing evidence of treatment failure by adopting strategies to increase compliance to the existing monotherapies. Major interventions were the prepackaging of these monotherapies and the introduction of home management of malaria. Furthermore, there was an extensive interaction between WHO, NMEP, the PMG and the regulatory authorities in which consensus was reached that age-specific antimalarial drugs should become available. While the PMG went ahead to implement these changes, the need for ACT as a strategic cornerstone for the treatment of uncomplicated malaria was gaining momentum at international levels.” (Interview Expert1).*

Therapeutic efficacy studies (TESs) conducted in 2002 showed that the corrected adequate clinical and parasitological response (ACPR) for chloroquine and SP in Nigeria were abysmally low at 34.7% and 57.4% respectively [40]. *An immersed expert* recalled these worrying developments, *“…The NMEP identified the next stage of the decision-making of selecting the most appropriate ACT. This was reported as the decision of another stakeholder meeting held in 2004.” (Interview Expert1).* The communiqué of that meeting emphasized the prevalence of both chloroquine and SP resistance in the country, the proven efficacies of the new ACT as reported from other countries and the need to conduct a local TES on the candidate artemisinin-based combinations (artemether-lumefantrine (AL) and artesunate-amodiaquine (ASAQ)) to inform programmatic deployment. Hence, another TES was conducted in 2004 that indicated efficacies above 90% for both artemisinin-based combinations for the treatment of uncomplicated falciparum malaria [40]. Due to the availability of AL as a co-formulated drug and high level of tolerability, the country adopted AL as the new first-line anti-malarial, while ASAQ, though a co-first line was reserved as an alternative or provided only by some donors as a cheaper alternative to support care in some areas of the country [40]. The donors were not the drivers of the adoption of the policy. However, considering the financial gap in making AL available everywhere in the country, other partners contributed and tried to stretch their contribution by using the relative cheaper but equally effective ASAQ.

*An immersed expert* narrated *“…this decision of the country, despite its intent, did not engage the PMG at any point. It was therefore regarded as a betrayal of the trust that has been built with the PMG. They complained of significant economic loss from the investments made to re-tool and re-register the newly prepackaged monotherapies. The result was that of a significant push back through intense advertisement of monotherapies by the PMG and the lack of uniformity in malaria treatment messaging. The FMoH had to set up a special committee to interphase and handle the change of management process. These issues delayed implementation of ACT use despite the relative early adoption of the ACT policies” (Interview Expert 1).* There is devolution of health system into federal, state and local levels thereby making each level autonomous policy maker and requiring concurrence of policy adoption. Because the States were not adequately engaged by the FMoH and NMEP during the policy adoption in Nigeria, there was delay in their concurrence to the policy as states below “…*furthermore, several states did not include ACTs in their essential medicine lists and could therefore not invest in the ACTs. In addition, the National Health Insurance Scheme (NHIS) also continued to recommend the use of monotherapies because the capitation fees being charged was significantly related to cost of monotherapies since fever/malaria was the commonest reason for outpatient consultations” (Interview Expert1).* The NHIS which enrolled over 5% of the formal sector in the country with just 10% co-payment from the enrollees generates its drugs and services list from which prescribers are allowed for standard benefit package [49]. As stated by the respondent, inadequate engagement of NHIS by the NMEP and FMoH allowed for continued use of monotherapy anti-malarials in the NHIS drugs list, for a few years after the adoption of ACT.

The important lesson learned is that the PMG and NHIS were not adequately engaged by the FMoH and NMEP as stakeholders in the policy change procedure, leading to inefficient deployment. The same applied for marketers and distributors who had previously invested and engaged in alternative therapies and were not compensated for these investments. There was a long delay before the prescribers had awareness of the policy change because of poor downstream communication to these stakeholder groups.

### ACT policy implementation and lessons learned

Following the adoption of the new combination therapy, Nigeria rolled out implementation of ACT immediately. This was shorter than the average time-lag of 12–18 months between policy adoption and implementation as reported from endemic regions [50, 51]. Although Nigeria encountered some delays in evidence uptake until policy adoption (between 2001 and 2005), this was compensated for by zeal and political will to implement ACT as new treatment regimen due to local evidence from local TES conducted in 2004 [40]. This was in turn reinforced by funding from the global developmental partners [42]. However, there was an immediate push-back among the pharmaceutical companies who had hitherto invested heavily on age-specific pre-packaged chloroquine [52, 53] and those already co-formulating SP for a large demand for Eastern Africa [44].

The implementation strategies were also met with resistance from suppliers, distributors, prescribers, and the end-users. Multiple reasons were adduced for the initial apathy [54]. According to the clinicians interviewed, the antipyretic effects of chloroquine that gave immediate relief to the patients and a sense of effectiveness to the prescribers encouraged resistance to the policy change (IDI Clinician1) and the suppliers created a sustained demand for chloroquine. A policy maker mentioned *“…the resistance from the private sector who probably at that particular time have invested their resources to produce these monotherapy drugs and were not carried along when this policy of changing to ACT combination drugs came, they were not properly orientated.” (IDI POLREG05).*

Other respondents noted that stakeholder engagement with regulatory agencies such as NAFDAC by the policy makers using the evidence is essential for the adoption of anti-malarial treatments. Failure to engage them could lead to delays in the adoption process. A policy maker mentioned* “The NAFDAC plays important role in regulation of the newly introduced antimalaria drugs in the country. So, NAFDAC is at the point of entry of any product, irrespective of which program is advancing for such products, NAFDAC need to accredit such drugs. And if that is not done, then there is every tendency that such drug will not be allowed at the program level.” (IDI POLREG02).*

Another factor was the cost of chloroquine compared to ACT especially among private prescribers and the suppliers. In low-income settings like Nigeria, affordability, and accessibility to ACT is crucial. The global supply and demand shortfall for ACT became an immediate burden that warranted WHO, UNICEF and other developmental partners outsourcing the procurement of ACT for the public sectors in the endemic regions, Nigeria inclusive [41, 42]. Evidence from Nigeria in the early period of policy adoption and implementation revealed mixed reports on the problematic transition to ACT in Nigeria particularly on the trust and initial skepticism to the ACT by the prescribers and the end-users. Some end-users and prescribers interviewed cited challenges related to prescribers’ distrust in ACT efficacy compared to the well-known chloroquine, while patients reported discomforting side-effects to amodiaquine.

The post-adoption phase of ACT deployment in Nigeria witnessed more developmental partner support for malaria control activities, robust data generation from efficacy studies and subsequent programme evidence. The ACT case-management strengthened with backup of this evidence then became established in treatment guidelines [35, 40]. One major fall-out of the post-adoption was the monopoly of the ACT supply by the pharmaceutical industry while global efforts were compelled to patronize the monopoly. This was detrimental to the survival of the local pharmaceutical industries who had previously been major stakeholders in the policy change adoption in Nigeria. To solve this problem and to reduce the high costs of ACTs among the populations in the endemic countries, the global efforts in 2008 developed the Affordable Medicine Facility-malaria (AMFm) which was piloted in seven African countries including Nigeria [55]. This policy attempted to supply ACT at a more affordable rate to the public and private systems through the principle of first line buyers who bought at a highly subsidized rate, and they were allowed a limited profit margin to make the ACTs affordable. After the pilot phase of the AMFm, the scale-up was implemented under the nomenclature of Private Sector Co-payment Mechanism (PSCM). The operational model was essentially similar except that there was increasing prettification of the first line buyers in the private sector responsible for a fraction of the cost of ACT [56].

The PSCM intervention had an initial positive impact on availability of ACT, which increased significantly over the period of implementation [56]. The impact was observed particularly among PPMVs with associated increased access to ACT by the poor households [56]. But similar to previous subsidized public health interventions, the programmes were not sustainable [55]. Another limitation was that important stakeholders in the downstream of ACT policy uptake like private prescribers, PPMVS and other distributors were completely neglected in the subsidy regime [57]. Therefore, the AMFm/PSCM intervention was discontinued. Nevertheless, AMFm/PSCM still leaves a regulated supply chain for malaria in Nigeria including a stabilized ACT cost. The lesson learned was that private sector engagement was inadequate since negotiated ACTs through the AMFm/PSCM arrangement were largely available through the public sector. As with PMG, the private sector was further hindered following the sudden withdrawal of both the AMFm and PSCM. Stakeholder groups reported that inadequate private stakeholder engagement at all levels led to the poor uptake of the policy at the initial phase. They suggested that these should be addressed to support the adoption of future anti-malarial treatments: *“To ensure that all important stakeholders especially the private sectors and end-users are involved in such important public health intervention like AMFm and PSCM, we need to support it with advocacy, communication, mobilization, and sensitization at the early phase. So, if we just deploy without following it up or without backing it up, we know the Behavioural Change Communication (BCC) component of our general attitude is difficult to attain.” (IDI POLREG03).*

### Moving forward and recommendation

Following the general principles, there is need for stakeholders’ identification/mapping, identification of the policy issues and purpose to engage the stakeholders [46]. Thereafter, strategies of engagement that take into consideration the local and socio-cultural peculiarities are important for an indigenous disease like malaria. Lastly, predetermining measurable policy adoption outcomes and achievable benefits must be set.

For engagement to be very effective, some respondents suggested that the content of messaging and communication regarding the rationale for a change in policy has to be well adapted to the peculiarities of the stakeholders. “*They will be able to tell people the benefits of the [new] drugs.” (End-user FGD 01). “…we give them health education about the drug, we should train the community….“ (Suppl FGD 01)* Some prescribers suggested that the policy change in anti-malarial should be contained in the treatment guidelines for the health workers. While this could be effective for the public health facilities, past experience has shown poor effectiveness of guidelines for the private health facilities. “…*if public facilities are very much aware of the change in policy, the reasons for the change and other information about the new drugs as detailed in the treatment guidelines, most public facilities would strictly go by the guidelines because education is done on various aspects of health and malaria being one of them but for the private sector because they are profit driven many would want to give their clients the satisfaction, there may be the tendency for them not to go by the guideline strictly” (IDI Healthworker1 Pub).*

A number of communication channels were recommended as effective strategies for communicating the rationale for a change in policy. Below are extracts of some of the narratives from the stakeholders;

“The government should help us announce very well on the radio and when something like this is available, they should inform the king of the community. He will find a way to disseminate information either through mosque or church when the government announce on the radio. They should tell the King and he will inform the people”. (End-user FGD 05).

“…… in the olden times, we used the town crier…. so, I think we can use the same means. Each community will decide what to do. Some use mosques, some use churches, they will make the announcement there”. (End-user FGD 05).

“If you can embark on door-to-door awareness they will readily accept it”. (End-user FGD 06).

“Whenever you identify the leaders within that community, there's always a particular leader in each section of the community, they will accept it”. (End-user FGD 04).

“Maybe Pastors of the church, the Imam and Alfas and the schools and the clinic, the health workers they would also play a very big role to make sure that the community accepts the drugs” (IDI End-user 03).

Several stakeholders highlighted the importance of making training of health workers an integral part of the deployment process. They suggested that building the capacity of health workers would enable them to provide the right information about rationale for deployment of any new treatments at the community level. Policy makers suggested “*People should be enlightened about the drug; they should know the composition of the drug and know the side effects too so that there will be no resistance.” (IDI POLREG08). Another regulatory authority mentioned “They should train them about the new drug that is coming, the composition of the drug, we can hold a seminar or workshop to boost their capacity.” (IDI REG 08).* These views were also shared by suppliers “*There should be seminar for health workers, they should pay people for attending the seminar.” (Suppl FGD 02).*

However, the health workers gave a concise account of what information the health workers need. *“…we have always trained prescribers on clinical things, you know when there is a new drug, we talk to them about the drug, the resistance, how they should give it, I think our former approach is boring for the new generation of clinicians who are on Instagram, Twitter and other social media, (IDI Healthworker 2 Pub).* One expert added a general recommendation on engagement of stakeholders emanating from anti-malarial policy change evidence which is sourced locally as follows;* “by having stakeholders perspective even from the beginning of evidence generation and for a country like Nigeria when it comes to marketing the challenge is that people go elsewhere for ease of the study when they come to Nigeria they want to market, Nigerians feel left out that other people enjoy the benefit of research, they would have wanted that there is some investment in the country and in any case once evidence is generated from the country it is easier to communicate and I must say the country presently knows how to take decisions from evidence which contains all the information that all the stakeholders need……….” (Interview Expert 2).*

The distributors and prescribers of anti-malarials (especially in the private settings) are identified from the interviews as important stakeholders for acceptability of the new policy change and accessibility to the new drugs. *“most engagements happens in the public sector and it is drug companies trying to push it to the private sector (prescribers and distributors), people (end-users) who come into private sector are large, d so they have to involve the mothers because they are the core care givers and end-users when it comes to malaria, they must know about it,….. the largest group in the drug sector are the chemists not even the pharmacists, they are the ones in every street corner, so they are also very important. The communication about the new policy must come in different languages, they must come in different innovative ways if it must be effective “(IDI Healthworker 5 Private).*

Engagement with end-users is required for successful adoption of any new anti-malarial policy as summed up by a respondent: *“There are gatekeepers within the community; it could be traditional ruler, it could be a philanthropist within that community that is well respected and that may have contributed to the development of that environment in one way or the other, so they are key.” (IDI REG01). The perspectives of end-users suggested that there are several reasons why engagement should be an integral part of the having adequate knowledge of any new antimalarial treatment. End-users stated reasons for the engagement: “if they are informed before that if you take this drug, this is the problem, then they will not worry” (End-user FGD 01). Another end-user buttressed this: “Since we all know that initially, so definitely every drug that will be highly effective must come with side effects. So, they should tell the community this is the side effect of this drug…….” (End-user FGD 01).* Most end-users suggested that engagement processes that seeks to address issues related to health-seeking behaviour should be adopted: *“So I think one of the strategies is to have an early engagement of the local communities, their health-seeking behaviour ultimately influences whatever you are doing, we can reduce all of those obstacles.” (End-User IDI01,). Other respondents during discussion gave insight into drug non-adherence because of lack of end-user engagement “I don’t normally complete the dosage. Once I feel much better, I stop using it.” (End-user FGD 04). “…well we have this mentality of being doctors in the house before going to the medical doctor, we treat with paracetamol and when the patient is not responding we go to the counter to get some malaria drugs like Lonart, so that is what we do primarily but if there is no response we now go to the hospital to see medical doctor.” (End-user IDI 03).*

Policy makers and regulators (e.g. NAFDAC) should be engaged by the NMEP and FMoH through adequate, convincing, and locally acceptable evidence from research and efficacy trial from the country or similar setting. This will give credibility and fidelity to the new anti-malarial. As narrated in the policy change era to ACT, the private sector distributors, marketers, and prescribers will play key roles (as shown in the framework) if engaged earlier. Engagement of health practitioners is essential for a transition to a new anti-malarial drug or treatment strategy. Several respondents anticipated challenges in private sector, where retailers and prescribers are often guided by patient demand rather than treatment guidelines. Especially in the private sector. This was considered a potential barrier to future adoption and would require active engagement of the private sector stakeholders. The same applies to a large number of informal retailers, as ‘over the counter’ prescription without expert consultation remains common in Nigeria [58]. Some suppliers mentioned that retailers and prescribers are not always aware of the magnitude of drug resistance, its causes and risks, and its implications. Therefore, providing training and information was considered important by some respondents. Such information campaigns should begin with awareness of the threat of anti-malarial drug resistance and the risks involved. They should be educated on the benefit of deploying new drugs with a view to delay or prevent multidrug resistance: *“So, these are lessons learnt that moving forward, if there’s anything of such a nature, and more importantly, when we are at this stage, this is the time we even need to start engaging the patient at the community.” (IDI POLREG05).*

Learning from the experiences of the transition from monotherapies to ACT, all stakeholder groups shared the view that implementation programs and behavior change initiatives are important to engage practitioners and patients in a prospective transition to a new therapy. The NMEP, under coordination of the Ministry of Health (MoH), was considered the most credible party to coordinate such initiatives in Nigeria. Some policy makers gave examples of models of engagement that have worked in local communities in the provision of health services that could be adopted for engagement regarding the introduction of new anti-malarials: *“when we initially introduced the ACT, there's one we called role model. In deploying some of these drugs, role model has key roles to play because if you look at it, it takes a patient like 100 min to move from his house to facility. But within a household, if we have a respected role model within the community, people will believe in him or her than the people that they, maybe, see them once in blue moon whenever they go to the hospital… but these individuals… we need to look at how we engage them.” (IDI POLREG08).*

### Proposed framework for stakeholder engagement in adoption of new anti-malarials or treatment strategies

From the foregoing, we propose a framework for effective stakeholder engagement for future adoption of new anti-malarial treatments or treatment strategies in Nigeria. The framework covers the pathway from generating evidence to making the treatments accessible and affordable to end-users. It also addresses key elements and recommendations on who to engage, the content of engagement and what strategies would support effective engagement with various key stakeholders at different levels of a transition process. The triad of evidence, policy and implementation is envisioned in a well-coordinated stakeholder spectrum with active two-way interrelationships between the stakeholders which will be informed by critically outlined strategies [59]. Adopting the five pillars of IAP2, [46] a five-step approach to engage stakeholders was identified as shown below.

1. *Inform *Provide balanced and objective information on the policy change to the stakeholder in terms of the purpose, opportunities, and limitations of the policy.
2. *Consult* Obtain feedback from the stakeholder on the assessment and their understanding of the policy change with the view of the local alternatives, challenges, and decisions. This will improve the implementation and acceptability of the new policy.
3. *Involve* Work directly and together with the stakeholders to ensure that their concerns, aspirations and challenges are understood and taken into consideration.
4. *Collaborate* Reiterate this is a partnership relationship with the stakeholders to take the policy change adoption decision together and to identify preferred best solutions.
5. *Empower *Place the policy adoption in the hands of the stakeholder for sustainable implementation and feedback.

Adapting these to the Nigeria case of anti-malarial policy change adoption, critical stakeholders as shown in the conceptual framework (Fig. 1) were identified. Then,the approaches above was applied to the framework in Fig. 1 guided by the lessons learnt from the interviews, policy and programme review of the NMEP in Nigeria.

#### Evidence provider

Providing credible and locally acceptable evidence on anti-malarial policy change is important to all the stakeholders in the drug demand and provision in Nigeria. The WHO played this role and coordinates all other malaria control efforts globally and especially in the malaria endemic regions of Africa. In turn, the country malarial control and elimination programmes (NMEP) and ministries of health are central to the in-country evidence provision. The WHO inform the country malaria control programmes, ministries and other in-country stakeholders of efficacy trials, routine surveillance and global trends. Informing evidence that will be locally acceptable and credible is important for the evidence to influence policy change. Consultations at global, regional, national and local levels are cardinal to the country buy-in. During consultations, concerns and specific peculiarities regarding the anti-malarial policy change are addressed. Effective consultations will foster partnership relationship with the stakeholders for policy change adoption by involving them in decision-making. Fostering collaboration by the evidence provider with other stakeholders is seamless when the stakeholders at the apex of diseases control in the country are informed, consulted and involved. The support of evidence providers like WHO, the US Presidents Malaria Initiative (PMI), malaria consortium, other global partners, and researchers in the country to empower the country control programme, ministries and stakeholders to understand the evidence on policy change and the various options and strategies available will strengthen the capacity to implement the policy.

#### Policy-makers

These stakeholders make policies on anti-malarial and other malaria control activities and strategies. They are the most important stakeholders since they have to accept the evidence for policy change. They also have the role to inform the other stakeholders in the policy implementation spectrum. Learning from the Nigeria adoption of ACT, previous anti-malarial policy change in the country witnessed a delay because of inability of the Nigerian policy makers to accept the evidence. However, the Nigeria policy maker (FMoH representing the Nigerian government) changed anti-malarial policy the 2004 TET conducted in the country established loss of monotherapy anti-malarial efficacy in the country. The policy makers must inform, consult and involve the country’s programme agencies including the regulators, prescribers, distributors and end-users. They must have input in the policy and draw up the implementation plans early. At this stage, other partnering stakeholders on anti-malarial control must be involved and consulted. The policy must have a plan for empowering the stakeholders in the implementation (distributors, prescribers and end-users) of anti-malarial policy.

#### Regulators

These stakeholders are responsible for the market and ethical aspect of the anti-malarial deployment in the country. In Nigeria, NAFDAC, Standard Organization of Nigeria (SON) and other professional bodies regulators play major roles. They are the middle link between policy adoption and policy implementation. Early involvement by information, consultation and collaboration of the regulators from the evidence generation and alternate anti-malarial trials stage guarantees support from the regulators. For instance, all the drugs on trial or are dispensed in the country are regulated and approved by the NAFDAC. Early involvement in the policy change will empower these regulatory agencies. From the interviews, the regulators (NAFDAC) observed delay in the previous anti-malarial policy change in the country and recommended for early involvement and collaboration from both the evidence generators and policy makers.

#### Marketers/distributors/manufacturers

This group constitute critical elements in the supply chain system, providing the linkage between anti-malarial supply and consumption. They are a majorly private, for-profit group with financial interest and profits. From the Nigerian experience, the group (PMG and the PPMVs) were major obstacles to the previous anti-malarial policy change. Reasons adduced were lack of early involvement and collaborations in addition to high cost of ACT compared to monotherapies. Addressing the push back from PMG led to intensified collaboration and empowering of the manufacturers and distributors through the co-payment systems of the AMFm and PSCM strategies. Going forward, early engagement of this group of stakeholders, through adequate information on the evidence, wide consultation of the policy change, involvement in the decision making of the policy change implementation on how it affects their supply chain and business; and areas of collaboration including empowerment plans will encourage full cooperation of this group.

#### Prescribers/dispensers

These are the frontline stakeholders in the inter-phase between final consumers of anti-malarials. They implement the policy change and are trusted more by the end-users group. This group also comprise of large private and informal sectors that prescribe and dispense drugs. A large proportion of Nigerian population patronizes the private/informal sector. The reviews from this study established that the prescribers (especially private/informal sectors) were reluctant to implement previous policy change in Nigeria due to perceived impact on their financial gains and lack of information on the policy. To address future anti-malarial deployment from a policy change, this group needs to be adequately informed of the evidence of new anti-malarials, including adequate training, involvement, and consultation on the best practices, and cost-benefits for the deployment. The collaboration with this group on efficacy trials, surveillance, and post-market trials of anti-malarials is important for continuous credibility and acceptance of the new anti-malarial drugs. This stakeholder group also require locally generated evidence on the policy change regarding the new anti-malarial because of the end-users’ trust is vested on them.

#### End-users

This stakeholder’s group is the ultimate target of any policy change and unfortunately the least informed of the policy. The end-users are diverse and dynamic. They are organized in sub-groups at times with leadership structures. In Nigeria, end-users are from communities with religious, traditional, political and social leaders. These leadership structures are important for mobilization, awareness creation and collaboration. Another important end-user category are the care-givers especially of the children under 5 years. These are mostly mothers and usually take decisions on behalf of their children. In the past policy change on anti-malarials, the end-users were not adequately engaged. The lack of information of ACT resulted in the persistent use of monotherapies till date and the continuous provision of same by the marketers and prescribers (as a demand feedback). The future anti-malarial deployment should focus attention on informing, consulting and involving the end-users on the need for the policy change and the properties including the cost and side effects of the new anti-malarial. Several media of engaging end-users have been described in the previous sections. Finally, the private sector prescriber and distributors are cardinal to end-users information and involvement in the policy change. Therefore, future policy change should engage private sector early and adequately to achieve a maximum anti-malarial adoption.

## Conclusion

This case study reviewed the historical evolution of anti-malarial drug policy change in Nigeria and used empirical data to explore the stakeholders’ engagement experience during the policy change. It adopted the five pillars of International Association of Public Participation (IAP2) to synthesize stakeholder engagement spectra and proposed a framework for stakeholder engagement in adoption of new anti-malarials or treatment strategies in the future. It identified the importance of evidence provision from trusted and credible stakeholders to engage the national policy makers and regulators in the malaria control and elimination. The importance of early engagement of Marketers/Distributors/Manufacturers, prescribers and end-users groups is highlighted as critical to the successful adoption of any new treatment strategy for malaria.

## Data Availability

There are ethical and legal restrictions to sharing our data publicly but they are available upon request from MORU Data Access Committee (https://www.tropmedres.ac/units/moru-bangkok/bioethics-engagement/data-sharing). Most interviews are directly traceable to individual identities and therefore cannot share the interview data without releasing the identities of the respondents. In each interview, respondents introduced themselves and spoke about their direct (working) environment. Moreover, the topics discussed and responses to questions, could be directly linked to their job positions and affiliation, especially with higher level policy and regulatory officials. We guaranteed full anonymity to the respondents prior to data collection and therefore sharing the dataset without restrictions would be unethical.

## Abbreviations

ACPR: Adequate clinical and parasitological response
ACT: Artemisinin-based combination therapy
AL: Artemether-lumefantrine
AMFm: Affordable medicine facility-malaria
ASAQ: Artesunate-amodiaquine
BCC: Behavioural change communication
FGD: Focus group discussion
FMoH: Federal Ministry of Health
IAP2: International Association of Public Participation
IDI: In-depth interview
IPT: Intermittent preventive treatment
IRB: Institutional review board
NAFDAC: National Agency for Food and Drug Administration and Control
NHIS: National Health Insurance Scheme
NMEP: National malaria elimination programme
PMG: Pharmaceutical manufacturing group
PPMVs: Proprietary patent medicine vendors
PSCM: Private sector co-payment mechanism
SMC: Seasonal malarial chemoprevention
SON: Standard Organization of Nigeria
SP: Sulfadoxine-pyrimethamine
TACT: Triple artemisinin-based combination therapy
TES: Therapeutic efficacy studies
UNICEF: United Nation Children’s Fund
WHO: World Health Organization

## References

[1] WHO (2016). Global technical strategy for malaria 2016–2030.

[2] Bosman A, Delacollette C, Olumese P, Ridley R, Shretta AR (2001). The use of antimalarial drugs: report of an informal consultation.

[3] Humphreys K, Piot P (2012). Scientific evidence alone is not sufficient basis for health policy. BMJ.

[4] Webster J, Hoyt J, Diarra S, Manda-Taylor L, Okoth G, Achan J (2020). Adoption of evidence-based global policies at the national level: Intermittent preventive treatment for malaria in pregnancy and first trimester treatment in Kenya, Malawi, Mali and the Gambia. Health Policy Plan.

[5] de Haan F, Moors EHM, Dondorp AM, Boon WPC (2021). Market formation in a global health transition. Environ Innov Soc Transit.

[6] Phok S, Phanalasy S, Thein ST, Likhitsup A (2017). Private sector opportunities and threats to achieving malaria elimination in the greater Mekong Subregion: results from malaria outlet surveys in Cambodia, the Lao PDR, Myanmar, and Thailand. Malar J.

[7] Toe P, Journal M, Toe LP, Dicko B, Linga R, Barry N (2022). Operationalizing stakeholder engagement for gene drive research in malaria elimination in Africa — translating guidance into practice. Malar J.

[8] Adeyemo AO, Aborode AT, Bello MA, Obianuju AF, Hasan MM, Kehinde DO (2022). Malaria vaccine: the lasting solution to malaria burden in Africa. Ann Med Surg.

[9] Phyo AP, Nosten F, Manguin S, Dev V (2018). The artemisinin resistance in Southeast Asia: an imminent global threat to malaria elimination. Towards Malaria Elimination.

[10] White NJ (2021). Emergence of artemisinin-resistant *Plasmodium falciparum* in East Africa. N Engl J Med.

[11] Balikagala B, Fukuda N, Ikeda M, Katuro OT, Tachibana S-I, Yamauchi M (2021). Evidence of artemisinin-resistant malaria in Africa. N Engl J Med.

[12] Uwimana A, Umulisa N, Venkatesan M, Svigel SS, Zhou Z, Munyaneza T (2021). Association of *Plasmodium falciparum* kelch13 R561H genotypes with delayed parasite clearance in Rwanda. Lancet Infect Dis.

[13] WHO. Strategy to respond to antimalarial drug resistance in Africa. WHO/UCN/GMP/202204. 2022. https://cdn.who.int/media/docs/default-source/malaria/who-antimalarial-drug-resistance-strategy-for-consultation.pdf?sfvrsn=9d4eaa0_6

[14] Trape JF (2001). The public health impact of chloroquine resistance in Africa. Am J Trop Med Hyg..

[15] Sowunmi A, Akano K, Ntadom G, Ayede AI, Ibironke FO, Aderoyeje T (2017). Therapeutic efficacy and effects of artemisinin-based combination treatments on uncomplicated *Plasmodium falciparum* malaria -associated anaemia in Nigerian children during seven years of adoption as first-line treatments. Infect Dis Poverty.

[16] WHO-CDS-GMP.  (2018). Artemisinin resistance and artemisinin-based combination therapy efficacy.

[17] Dorkenoo AM, Yehadji D, Agbo YM, Layibo Y, Agbeko F, Adjeloh P (2016). Therapeutic efficacy trial of artemisinin-based combination therapy for the treatment of uncomplicated malaria and investigation of mutations in k13 propeller domain in Togo, 2012–2013. Malar J.

[18] Gansané A, Moriarty LF, Ménard D, Yerbanga I, Ouedraogo E, Sondo P (2021). Anti-malarial efficacy and resistance monitoring of artemether-lumefantrine and dihydroartemisinin-piperaquine shows inadequate efficacy in children in Burkina Faso, 2017–2018. Malar J.

[19] Dimbu PR, Horth R, Cândido ALM, Ferreira CM, Caquece F, Garcia LEA (2021). Continued low efficacy of artemether-lumefantrine in Angola in 2019. Antimicrob Agents Chemother.

[20] Rasmussen C, Ringwald P (2021). Is there evidence of anti-malarial multidrug resistance in Burkina Faso?. Malar J.

[21] Rasmussen C, Ringwald P (2021). Continued low efficacy of artemether-lumefantrine in Angola?. Antimicrob Agents Chemother.

[22] Moriarty LF, Nkoli PM, Likwela JL, Mulopo PM, Sompwe EM, Rika M (2021). Therapeutic efficacy of artemisinin-based combination therapies in Democratic Republic of the Congo and investigation of molecular markers of antimalarial resistance. Am J Trop Med Hyg.

[23] Tse EG, Korsik M, Todd MH (2019). The past, present and future of anti-malarial medicines. Malar J.

[24] Chen I, Hsiang MS (2022). Triple artemisinin-based combination therapies for malaria : a timely solution to counter antimalarial drug resistance. Lancet Infect Dis.

[25] van der Pluijm RW, Amaratunga C, Dhorda M, Dondorp AM (2021). Triple artemisinin-based combination therapies for malaria—a new paradigm?. Trends Parasitol.

[26] de Haan F, Bolarinwa OA, Guissou R, Tou F, Tindana P, Boon WPC (2021). To what extent are the antimalarial markets in African countries ready for a transition to triple artemisinin-based combination therapies?. PLoS One..

[27] Tindana P, Guissou R, Bolarinwa OA, Tou F, de Haan F, Dhorda M (2022). Ethical considerations in deploying triple artemisinin-based combination therapies for malaria : an analysis of stakeholders ’ perspectives in Burkina Faso and Nigeria. PLoS ONE.

[28] Tindana P, de Haan F, Amaratunga C, Dhorda M, van der Pluijm RW, Dondorp AM (2021). Deploying triple artemisinin-based combination therapy (TACT) for malaria treatment in Africa: ethical and practical considerations. Malar J.

[29] de Haan F, Boon WPC, Amaratunga C, Dondorp AM (2022). Expert perspectives on the introduction of Triple Artemisinin-based Combination Therapies (TACTs) in Southeast Asia: a Delphi study. BMC Public Health.

[30] van der Pluijm RW, Tripura R, Hoglund RM, Pyae Phyo A, Lek D, Ul Islam A (2020). Triple artemisinin-based combination therapies versus artemisinin-based combination therapies for uncomplicated *Plasmodium falciparum* malaria: a multicentre, open-label, randomised clinical trial. Lancet.

[31] Tindana P, de Haan F, Mokuolu OA, Guissou R, Bolarinwa OA, Ouedraogo JB (2021). Ethical, regulatory and market related aspects of deploying triple artemisinin-based combination therapies for malaria treatment in Africa: a study protocol. Wellcome Open Res.

[32] WHO. World Malaria Report (2020). 20 years of global years and challenges.

[33] Federal Ministry of Health. National Malaria control programme strategic plan 2009–2013: a road map for malaria control in Nigeria Abuja; 2013. Available from: https://extranet.who.int/countryplanningcycles/sites/default/files/planning_cycle_repository/nigeria/nigeria_draft_malaria_strategic_plan_2009-2013.pdf.

[34] ACTwatch’Group’and’SFH. ACTwatch’Study’Reference’Document: “The’Federal’Republic’of’Nigeria” Outlet’Survey’ 2015. Washington!DC:!PSI.; 2015. http://www.actwatch.info/sites/default/files/content/publications/attachments/Nigeria_2015 OS_Reference Document.pdf.

[35] FMoH. National Malaria Policy 2014–2020. [Internet]. Abuja, 2014;29. Available from: https://www.health.gov.ng/doc/NMEP-Strategic-Plan.pdf

[36] WHO. World malaria report 2021. Geneva; World Health Organization. 2021. https://www.who.int/teams/global-malaria-programme/reports/world-malaria-report-2021

[37] National Population Commission and ICF. Nigeria demographic and health survey. Abuja, Nigeria, and Rockville. Maryland, USA; 2018. 297–305 p. https://dhsprogram.com/publications/publication-fr359-dhs-final-reports.cfm

[38] World Bank Group. Incidence of malaria (per 1,000 population at risk)—Nigeria Data. https://data.worldbank.org/indicator/SH.MLR.INCD.P3?locations=NG

[39] National Population Commission. Federal Republic of Nigeria. Malaria Indicator Surveys in children 2–10 years. Abuja. https://dhsprogram.com/pubs/pdf/MIS41/MIS41.pdf

[40] Federal Ministry of Health. National antimalarial treatment guidelines. Abuja: 2005; 1–29.

[41] WHO. Briefing on Malaria Treatment Guidelines and artemisinin monotherapies - meeting_briefing19april.pdf. Geneva; World Health Organization. 2006. Available from. https://www.who.int/malaria/publications/atoz/meeting_briefing19april.pdf

[42] Bosman A, Mendis KN (2007). A major transition in malaria treatment: The adoption and deployment of artemisinin-based combination therapies. Am J Trop Med Hyg.

[43] Williams HA, Durrheim D, Shretta R (2004). The process of changing national malaria treatment policy: Lessons from country-level studies. Health Policy Plan.

[44] Nanyunja M, Nabyonga Orem J, Kato F, Kaggwa M, Katureebe C, Saweka J (2011). Malaria treatment policy change and implementation: the case of Uganda. Malar Res Treat.

[45] Amin AA, Zurovac D, Kangwana BB, Greenfield J, Otieno DN, Akhwale WS (2007). The challenges of changingnational drug policy to artemisinin-based combinations in Kenya. Malar J.

[46] Akwanalo C, Njuguna B, Mercer T, Pastakia SD, Mwangi A, Dick J (2019). Strategies for effective stakeholder engagement in strengthening referral networks for management of hypertension across health systems in Kenya. Glob Heart.

[47] Federal Ministry of Health. National Malaria Strategic Plan 2014 ‐ 2020. Policy document. Abuja. 2017. https://www.health.gov.ng/doc/NMEP-Strategic-Plan.pdf

[48] Abdullahi K, Muhammad S, Manga SB, Tunau IM (2003). Chloroquine-resistant *Plasmodium falciparum* in Sokoto. North Western Nigeria African J Biotechnol.

[49] National Health Insurance Scheme. National Health insurance scheme: membership Handbook. a guide for enrolees on the operations of the NHIS Formal Sector Programmes. Abuja; 2020. 7–10 p. https://www.nhis.gov.ng/?media_dl=2713

[50] Bosman A, Mendis KN (2007). A major transition in malaria treatment : the adoption and deployment of artemisinin-based combination therapies. Am J Trop Med Hyg.

[51] Mokuolu OA, Okoro EO, Ayetoro SO, Adewara AA (2007). Effect of artemisinin-based treatment policy on consumption pattern of antimalarials. Am J Trop Med Hyg.

[52] Goodman C, Brieger W, Unwin A, Mills A, Meek S, Greer G (2007). Medicine sellers and malaria treatment in sub-Saharan Africa: what do they do and how can their practice be improved?. Am J Trop Med Hyg.

[53] Brieger WR, Salako LA, Umeh RE, Agomo PU, Afolabi BM, Adeneye AK (2002). Promoting prepackaged drugs for prompt and appropriate treatment of febrile illnesses in rural Nigerian communities. Int Q Commun Health Educ.

[54] Yakasai AM, Hamza M, Dalhat MM, Bello M, Gadanya MA, Yaqub ZM (2015). Adherence to artemisinin-based combination therapy for the treatment of uncomplicated malaria: a systematic review and meta-analysis. J Trop Med.

[55] Talisuna AO, Adibaku S, Amojah CN, Amofah GK, Aubyn V, Dodoo A (2012). The affordable medicines facility-malaria—a success in peril. Malar J..

[56] Edwards HM, Sarwar R, Mahmud P, Emmanuel S, Maxwell K, Tibenderana JK (2022). The impact of the private sector co-payment mechanism (PSCM) on the private market for ACT in Nigeria: results of the 2018 cross-sectional outlet and household market surveys. Malar J.

[57] Ajayi IO, Soyannwo T, Akpa OM (2013). Awareness and utilization of affordable medicine facility-malaria among caregivers of under-five children in Ibadan North-West local government area. Oyo State Malar Res Treat.

[58] Durowade KA, Bolarinwa OA, Fenenga CJ, Akande TM (2018). Operations and roles of patent and proprietary medicine vendors in selected rural communities in Edu local government area, Kwara State, north-central Nigeria. J Commun Med Prim Health Care.

[59] Hutchinson E, Droti B, Gibb D, Chishinga N, Hoskins S, Phiri S (2011). Translating evidence into policy in low-income countries: lessons from co-trimoxazole preventive therapy. Bull World Health Organ.

